# Virulence of BotaniGard^®^ to Second Instar Brown Marmorated Stink Bug, *Halyomorpha halys* (Stål) (Heteroptera: Pentatomidae)

**DOI:** 10.3390/insects6020319

**Published:** 2015-04-09

**Authors:** Bruce L. Parker, Margaret Skinner, Svetlana Gouli, Vladimir Gouli, Jae Su Kim

**Affiliations:** 1 Entomology Research Laboratory, University of Vermont, 661 Spear Street, Burlington, VT 05405, USA; E-Mails: mskinner@uvm.edu (M.S.); sgouli@uvm.edu (S.G.); vgouli@uvm.edu (V.G.); 2 Department of Agricultural Biology, College of Agriculture & Life Sciences, Chonbuk National University, Jeonju 561-756, Korea; E-Mail: jskim10@jbnu.ac.kr

**Keywords:** brown marmorated stink bug, *Halyomorpha halys*, *Beauveria bassiana*, BotaniGard^®^

## Abstract

The brown marmorated stink bug, *Halyomorpha halys* (Stål) (BMSB) is an exotic invasive insect originating in East Asia, currently causing significant damage to fruits, vegetables and other crops throughout most of the Mid-Atlantic states of the U.S. It also is a nuisance pest, entering homes in the fall in search of suitable overwintering sites. Two formulations of BotaniGard^®^ with a strain of *Beauveria bassiana* (GHA) as the active ingredient were tested against second instar BMSB. Both the wettable powder and the emulsifiable suspension formulations were efficacious at 1 × 10^7^ conidia mL^−1^, causing 67%–80% mortality 9 days post treatment and 95%–100% after 12 days. The wettable powder formulation was slightly more efficacious.

## 1. Introduction

The brown marmorated stink bug, *Halyomorpha halys* (Stål) (Hemiptera: Pentatomidae) (BMSB) is an exotic invasive insect in the U.S. that has spread extensively from Pennsylvania, its first site of an accidental infestation [1]. It has been reported in 42 states in the U.S., including all those east of the Mississippi River and all those along the Pacific Coast [2,3]. Outside the U.S., it is also believed to be established in Canada, Switzerland and perhaps France [4]. Originating in East Asia (China, Japan, Korea, and Taiwan), it now causes significant damage in the U.S. to vegetables, fruits, ornamentals and other crops. Its importance has increased as it now has been identified as feeding on >300 different plants, many of which are economically important to U.S. agriculture. In addition, they are also a nuisance pest by entering homes in the fall to overwinter [5,6].

The efficacy of several chemical insecticides and their residual effects on adult BMSB have been assessed in fruit orchard field trials. Several of the products tested showed a high level of efficacy when BMSB were exposed to treated branches immediately after the pesticide application but, in general a residual effect 3 and 7 days post treatment was not observed [7]. In general, mortality rates among the BMSB were higher early in the growing season than in the mid- or late-season trials, suggesting that the overwintered adults were more susceptible to pesticides than those from the current growing season. In laboratory trials, residual mortality of 100% at 7 days after treatment was obtained for nine different insecticides when tested against nymphs [8].

In recent years, extensive research on biological control of BMSB has been conducted in the U.S. A wide variety of natural enemies have been identified in the native range of BMSB, and high rates of parasitism (63%–85%) in Asia were also reported [5]. In the U.S., surveys have been conducted to determine the impact of beneficial insects on BMSB, and while a broad array of species were identified, to date, none have demonstrated an ability to maintain this exotic pest below economically damaging levels. Limited research has been conducted to evaluate the potential of entomopathogenic fungi for use against BMSB populations. The entomopathogenic fungus, *Ophiocordyceps nutans*, was shown to be effective against BMSB in Japan [9]. The potential of entomopathogenic fungi against adult BMSB was evaluated in laboratory trials, and isolates of *Beauveria bassiana* and *Metarhizium anisopliae* caused significant mortality to the adults [10]. One of the most efficacious was a *B. bassiana* isolate, GHA, the active ingredient in the commercial product BotaniGard^®^ (Laverlam International Corp). Therefore, protection of the crop from damage by BMSB could be enhanced by targeting the early instars, thereby stopping further reproduction and preventing future generations. The purpose of the research reported herein was to determine the efficacy of two formulations of BotaniGard^®^ against second instar BMSB.

## 2. Experimental Section

Test insects (second instars) were obtained from a continuous lab culture of BMSB maintained at the University of Vermont Entomology Research Laboratory. Adult BMSB originally obtained from field-collected individuals in vegetables and fruits in New Jersey were reared in clear plastic cube cages (29.85 cm on each side) having three side panels with 24 × 24 cm plastic mesh screening (1.1 mm × 0.1 mm opening) and one side with a 16 cm diameter opening with a nylon sleeve to access the insects (BugDorm1, Catalog No. 1452, BioQuip Products, 2321 Gladwick Street, Rancho Dominguez, CA 90220, USA). To insure BMSB escape was prevented, each BugDorm was held within a larger cage (92 cm L × 61 cm W × 82 cm H) made with a PVC frame covered on all sides with nylon thrips-proof screening. The room in which the cages were held was lighted with four Sylvania Daylight Deluxe F40/DX T12 (6500K color temperature, 88 CRI) fluorescent bulbs with a 16 h light (L):8 h dark (D) photoperiod. Mean temperature in the cage was 23 °C (28 °C max, 18 °C min); RH was 30% (74% max, 7% min). They were cultured on carrots (*Daucus carota* var. *sativus* L.), green string beans (*Phaseolus vulgaris* L.) and sunflower seeds (*Helianthus annuus* L.). Water was provided for the insects by placing cotton balls moistened with tap water in the cage three times per week.

Two concentrations (5 × 10^6^ and 1 × 10^7^ conidia mL^−1^) of *Beauveria bassiana* (GHA strain) formulations were tested: BotaniGard^®^ 22WP (wettable powder), and BotaniGard^®^ ES (emulsifiable suspension), both obtained from BioWorks, Inc., Victor, NY, USA. In addition, two control treatments were tested: distilled water and untreated. Both formulations contained 11.3% fungal conidia as active ingredients and 88.7% inert material (2 × 10^10^ conidia g^−1^ or mL^−1^). The formulations were diluted to deliver the desired test concentrations. These concentrations were selected because in previous BMSB tests, they produced mortalities between 20% and 80% and were most appropriate for the evaluation of efficacy.

Prior to treatment, the viability of conidia in each suspension was determined using the method of Goettel and Inglis [11]. Suspensions with the required concentration of living spores were prepared based on the preliminary spore viability data. Based on visual inspection, healthy second-instar BMSB were collected in groups of five. Each group was placed in a separate test container (No. 23 Series Box, Dewitt Plastic Division, RPM Industries, Auburn, NY, USA) containing a sheet of filter paper. The test chamber measured 5.5 cm long × 6.5 cm wide × 2.8 cm deep. A 3.8 cm diameter hole was cut in the cover of the container and a piece of thrips-proof mesh cemented over it for ventilation. BMSB were sprayed with 1 mL of the treatment suspension with a hand-held atomizer. The atomizer apparatus was affixed to a plastic 33-mL vial. The atomizer was similar to those sold for small perfume bottles and pressure was created by depressing a finger on the pump section. Prior trials on spray deposition using glass cover slips in test containers held in the same type of test chamber showed that when the atomizer delivered 1 mL of the water-based spore suspension, 600–700 droplets were deposited per cm^2^. This amounted to around 550–600 conidia per mm^2^ for the 5 × 10^6^ concentration and 1100–1200 conidia per mm^2^ for the 1 × 10^7^ concentration.

After treatment, test containers were maintained on the laboratory bench at 22 ± 2 °C, 80%–85% RH and 12 h L:12 h D. Raw carrots, sunflower seeds and green beans were used as food and replaced every 3 days because when they dried out it was difficult for the test insects to feed on them. The old food was removed gently and discarded from each chamber after the new food was added. The insects clustered on the new food, eliminating the risk of them escaping.

Visual mortality counts were taken 3, 6, 9 and 12 days post-application. To avoid horizontal infection of other BMSB, at each period when mortality counts were made, dead individuals were removed, ensuring minimal disturbance to live individuals. The cadavers from both treated and control replicates were surface sterilized using standard methods [12]. Specifically they were dipped in 70% ethyl alcohol, rinsed in distilled water, dipped in a 1% sodium hypochlorite solution, and rinsed in distilled water.

After surface sterilization, cadavers from treated and control replicates were placed individually in sterile 3.5 cm diameter Petri dishes on filter paper moistened with distilled water and sporulation of *B. bassiana* recorded after 5 days, which indicated primary infection of the individuals. The Petri dishes with cadavers were held on a laboratory bench at 22 ± 2 °C, 80%–85% RH and 12 h L:12 h D. Each treatment including the controls was replicated three times within an experiment and the entire experiment was repeated four times on different occasions.

### Statistical Analysis

Prior to analysis, data were tested for normality and found to be normally distributed (*p* > 0.05). Data on the percentage of mortality among BMSB nymphs for the entire experimental period, combining results from all of the experiments, were analyzed using a general linear model (GLM), followed by Tukey’s honestly significant difference (HSD) for multiple comparisons. The analysis was conducted using SPSS ver. 17.1 at the 0.05 (α) level of significance [13].

## 3. Results and Discussion

**Figure 1:**
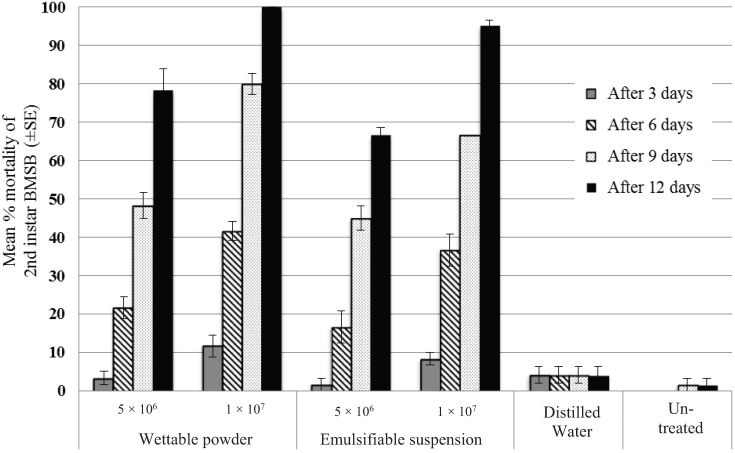
Mean percent mortality (± SE) of brown marmorated stink bug (BMSB) nymphs at 3, 6, 9 and 12 days after treatment with two concentrations of BotaniGard^®^ES or BotaniGard^®^ 22WP, distilled water, and untreated control. When error bars do not appear, there was no variability in the results.

When comparisons were made among the four separate experiments, differences in mortality levels were not significant, which allowed the combination of the full data set (*F*_3,192_ = 0.279, *p* = 0.840). Mortality of BMSB nymphs among the two control treatments (distilled water and untreated) ranged from 0 to 4.2% throughout the experimental period and were not significantly different (*p* = 0.633) (Figure 1). Mortality among BMSB nymphs treated with either of the fungal formulations at both concentrations was significantly higher than in the controls (*p* < 0.001).

For both formulation types, BMSB mortality was significantly greater for the high concentration treatment (1 × 10^7^) than the lower concentration (5 × 10^6^) (*F*_1,128_ = 127.1, *p* < 0.001). At the higher spore concentration, the wettable powder formulation exhibited greater efficacy than the emulsifiable suspension (mean difference = 6.67 ± 2.16, F_1,64_ = 11.529, *p* = 0.027). In contrast, for the low concentration, the two formulations showed similar levels of efficacy (mean difference = 5.42 ± 2.16, F_1,64_ = 4.568, *p* = 0.124). Mortality of the BMSB nymphs increased gradually over the 12 days after treatment with the wettable powder formulation reaching a maximum of around 78% for the low concentration and 100% for the higher concentration. For the emulsifiable suspension treatments, approximately 67% mortality was obtained for the low concentration compared to 95% for the higher concentration.

## 4. Conclusions

These bioassay results confirm that significant mortality of BMSB nymphs can occur following a spray treatment of the GHA strain of *B. bassiana* found in the commercial fungal-based product BotaniGard^®^. However, the wettable powder formulation provided slightly greater mortality than the emulsifiable suspension under controlled laboratory conditions. Somewhat similar results were obtained in bioassays conducted with the GHA strain (unformulated) against BMSB adults [10]. At 9 days after treatment with the GHA strain applied at a concentration of 1 × 10^7^, 80%–86% mortality was obtained for adults and immatures [10]. One hundred percent mortality was observed for both life stages 12 days after treatment. However, when a lower fungal concentration was tested (5 × 10^6^), at 9 days after treatment, 12% mortality was obtained in the trial with adults, compared with 48% for the immatures. At 12 days post-treatment, 60% mortality occurred for adults, and 88% for immatures [10]. This suggests that the immatures were more susceptible to infection than the adults. These results demonstrate the potential of entomopathogenic fungi as a biological control tool for managing this serious pest. Based on these results and those from our previous research, both the adult and immature stages of BMSB are impacted by treatment with the *B. bassiana* GHA strain. In addition, the immature stage is more sensitive to infection than the adults, therefore, growers may want to specifically target that stage to maximize reduction of the pest population. However, although these results show promise, trials are still needed to fully assess the efficacy of these fungal formulations under different environmental field conditions. In general, many comparisons of results from lab-based and field trials on the lethality of various chemical pesticides to BMSB have shown that mortality is considerably less in the field than in the laboratory [7].
